# Results of an attempt to reproduce the STAP phenomenon

**DOI:** 10.12688/f1000research.8731.2

**Published:** 2016-10-17

**Authors:** Shinichi Aizawa

**Affiliations:** 1 Scientific Validity Examination Team, RIKEN, Kobe, Japan

**Keywords:** STAP, iPSCs, ES, stem cells, chimera, Oct-GFP, pluripotency

## Abstract

In 2014, Obokata and colleagues reported their observation of a novel cell reprogramming phenomenon they named ‘stimulus-triggered acquisition of pluripotency’ (STAP). The most conclusive evidence for the pluripotency of so-called STAP cells was the purported ability of such cells to contribute to chimera formation. Here, I report the results of an attempt by Haruko Obokata to replicate the phenomenon under the supervision of the Scientific Validity Examination Team of RIKEN. In this follow-up study, putative STAP cells prepared by Haruko Obokata were injected into 1051 embryos, of which 591 were recovered. However, the injected cells made no significant contribution in any of the embryos that developed.

## Introduction

Induced pluripotent stem cells (iPSCs), first reported by Takahashi and Yamanaka using a combination of exogenous genetic factors, have transformed our understanding of the gene regulatory mechanisms underlying cellular pluripotency and differentiation ( Takahashi & Yamanaka, 2006). This discovery raised the possibility that cellular reprogramming may also be induced by activating endogenous pluripotency genes under certain conditions. In two reports published in *Nature* by Obokata *et al.* (2014a); Obokata *et al.* (2014b), the authors claimed to have observed how “external stimuli such as a transient low-pH stressor reprogram somatic cells into pluripotent cells,” which they referred to as the STAP phenomenon; subsequently, however, after multiple problems were found with the handling and presentation of the data in a pattern indicative of research misconduct, both papers were retracted.

The present article reports the results of a study conducted by Haruko Obokata in the RIKEN Center for Developmental Biology (CDB), which was designed to determine whether the STAP phenomenon was in fact reproducible. Obokata was permitted to perform this closely monitored study from July 14 to November 30, 2014 under my supervision as head of the Scientific Validity Examination Team, at the direction of the Head Office for Internal Reform organized by the RIKEN President. Unfortunately, I have been unable to contact her since the completion of the study, or to obtain her agreement to be listed as an author on this article. Nonetheless, given the extraordinary degree of attention and controversy the original STAP publications and research misconduct generated, I feel it is important to report the results of this investigation in the interests of clarifying the scientific record. In the Scientific Validity Examination Team, Hitoshi Niwa, one of the coauthors of the *Nature* papers, also conducted an independent examination of whether the STAP phenomenon was reproducible; the results of his examination have been reported previously ( Niwa, 2016).

The investigation reported here consisted of two types of experiments; preliminary ones conducted without supervision, and formal ones conducted in the presence of expert witnesses. There were no significant differences in the data generated in the preliminary and formal experiments, and all are included together in this report. The experiments were conducted in a new setting, not in the laboratory that Obokata had used for the previous studies described in the retracted *Nature* publications ( Obokata *et al.*, 2014a; Obokata *et al.*, 2014b). All reagents, materials, instruments, and experimental spaces were freshly furnished. Obokata was permitted to conduct experiments only in designated rooms. She prepared cell aggregates, but did not perform any of the subsequent analyses herself, other than observations of the cell aggregates by phase and fluorescence microscopy. Other members of the team conducted chimeric, FACS, qPCR and immunohistochemical analyses of the cell aggregates. In this report, I refer to the studies reported in the papers retracted ( Obokata *et al.*, 2014a; Obokata *et al.*, 2014b) as "the previous studies" for the sake of brevity. I also refer to the technical tips published by several authors of the original articles for details of the experimental procedure ( Obokata *et al.*, 2014c).

## Results

### Frequency of GFP-positive cells from spleen of *Oct-GFP* transgenic mice

Experiments were performed using a transgenic mouse line harboring *GFP* under an *Oct4* promoter ( Ohbo *et al.*, 2003); the line is the same as that used in the previous studies ( Obokata *et al.*, 2014a; Obokata *et al.*, 2014b). The mouse line has been maintained in C57BL/6 background in a homozygous state ( *B6 oct-gfp ^+/+^*). Spleens were dissected from homozygous newborn mice ( *B6 oct-gfp ^+/+^*; 6–8 days old) obtained by crossing a homozygous transgenic female ( *B6 oct-gfp ^+/+^*) with a homozygous transgenic male ( *B6 oct-gfp ^+/+^*), or from hemizygous newborn mice ( *F1 oct-gfp ^+/-^*; 6–8 days old) obtained by crossing a homozygous transgenic female ( *B6 oct-gfp ^+/+^*) with a wild type 129 male ( *129 oct-gfp ^-/-^*). Spleen cells were prepared as described previously ( Obokata *et al.*, 2014a; Obokata *et al.*, 2014c), but enrichment of CD45-positive cells by FACS sorting was omitted. The source of the cells used in these experiments were lymphocytes collected with Lympholyte following the manufacturer’s instructions (Cedarlane Laboratories, Ontario, Canada).

**Figure 1.**
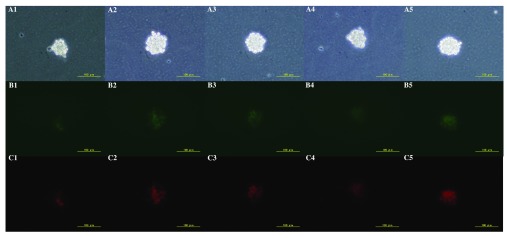
Examples of cell aggregates generated from *oct-gfp* transgenic spleen by low pH treatment. ( **A**) Phase contrast views of typical five cell aggregates, ( **B**) their green fluorescence and ( **C**) their red fluorescence.

The stress treatment evaluated was the low-pH condition; no other conditions, such as trituration, were examined. The low-pH conditions included not only the previously reported induction by HCl ( Obokata *et al.*, 2014a; Obokata *et al.*, 2014b; Obokata *et al.*, 2014c), but also that by ATP. Although not described in the previous reports, the ATP treatment had been used most frequently by Obokata *et al.*, and is described in their patent application regarding the STAP process (US Patent Application no.: 14/397,080). In brief, the low-pH condition was generated by suspending the 1×10 ^6^ cells in 494 μl HBSS (Hank’s Balanced Salt Solution), adding 6 μl 200 mM ATP, and incubating for 15 min at 37°C in CO _2_ incubator. The low-pH treated-cells were cultured for 6–8 days, and cell aggregates of 50–100 μm showing green fluorescence were identified (see Materials and methods). Table 1 gives the frequency of the cell aggregates identified by Haruko Obokata (see Materials and methods). The frequency of green fluorescent cell aggregates was slightly higher under ATP treatment than HCl treatment in the C57BL/6 background. However, no marked difference was found in the frequency of green fluorescent cell aggregates under either of the low-pH conditions (HCl or ATP) or genetic background of mice (C57BL/6 or F1 between C57BL/6 and 129). The observed frequency was approximately 10 green fluorescent cell aggregates per 10 ^6^ cells seeded; this was approximately 10-fold lower than that in the previous studies. Most green fluorescent cell aggregates also exhibited higher or lower degrees of red fluorescence ( Figure 1). No quantitative determination was made, but about one in three cell aggregates exhibited green fluorescence more intense than red fluorescence. Green fluorescent cell aggregates that exhibited no significant red fluorescence were rare.

**Table 1.** Frequency of cell aggregates from *oct-gfp* transgenic spleen after low pH treatment.

Mouse Background	Treatment	No. of Exp.	No. Fluorescent Cell Aggregates/10 ^6^ Cells Seeded		
			Average	Minimum	Maximum
C57BL/6	No treat.	9	0	0	0
	ATP	14	16	4	52
	HCl	11	10	3	23
F1 (C57BL/6×129)	No treat.	9	0	0	0
	ATP	13	12	1	30
	HCl	10	10	0	42


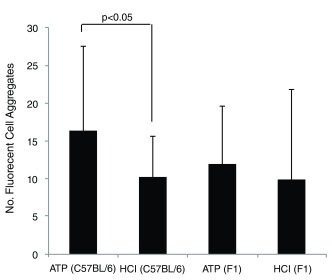


### Chimeric potency of ‘STAP’ cell aggregate

Chimera production was performed with spleens of a transgenic mouse line harboring *gfp* under a *CAG* promoter ( Okabe *et al.*, 1997) which were also maintained homozygously in C57BL/6 background ( *B6 cag-gfp ^+/+^*); this line is different from the one previously used ( Obokata *et al.*, 2014a; Obokata *et al.*, 2014b). Spleens were dissected from *cag-gfp* hemizygous newborn mice in C57BL/6 background ( *B6 cag-gfp ^+/-^*; 6–8 days old) obtained by crosses of wild-type C57BL/6 ( *B6 cag-gfp ^-/-^*) female with homozygous transgenic ( *B6 cag-gfp ^+/+^*) male, or from hemizygous newborn mice in F1 background between C57BL/6 and 129 ( *F1 cag-gfp ^+/-^*; 6–8 days old) obtained by crosses of homozygous transgenic female ( *B6 cag-gfp ^+/+^*) with wild-type 129 male ( *129 cag-gfp ^-/-^*). Cell aggregates of 50–100 μm were selected by their cluster morphology by Obokata and subjected to the chimeric assay. Chimeras were made by members of the Laboratory for Animal Resources and Genetic Engineering, CDB, with expertise in chimera production with ES cells ( http://www2.clst.riken.jp/arg/APDBN.html, http://www2.clst.riken.jp/arg/mutant_mice_generated_in_CDB.html)(present affiliation: Animal Resource Development Unit, Biosystem Dynamics Group, Division of Bio-Function Dynamics Imaging, Center for Life Science Technologies (CLST)).

The previous report indicated that the generation of chimeras using STAP cells involved a distinct technical approach ( Obokata *et al.*, 2014a): “Single cell dispersion by trypsinization, as it is done in the chimera production with ES cells, caused low chimaerism. STAP spherical colonies were cut into small pieces using a microknife under the microscope. Small clusters of the cells are then injected into blastocysts.” In the present study, cell aggregates were cut into small pieces by either glass capillary, laser beam (XY Clone: Nikko Hansen & Co., Osaka, Japan) or microsurgical knife (K-5310: FEATHER Safety Razor Co., Osaka, Japan) and were injected into a host embryo, either E2.5 8-cell stage or E3.5 blastocyst stage embryos of random-bred ICR (Charles River, Tokyo, Japan). Injected embryos were transplanted into the uterus of pseudopregnant females of the ICR strain, and recovered at E9.5 or E8.5 to judge the contribution of injected cells by GFP-green fluorescence ( Table 2). Notably, the previous study describes that small clusters of 'STAP' cells were injected into ‘E4.5 blastocysts’, and the next day, the chimeric blastocysts were transferred into pseudopregnant females ( Obokata *et al.*, 2014a).

**Table 2.** Chimera analysis of pluripotency.

Cell aggregates injected		No. host embryos ^3)^		Chimera analysis		No. chimeric embryos or mice
Genetic background ^1)^	Specifications ^2)^	morula	blastocyst	stage	No. developed	
C57BL/6	glass capillary	156	ー	E9.5	107	0
		ー	226	E9.5	117	0
		ー	66 ^4)^	E9.5	27	0
F1(C57BL/6x129)		54	ー	E9.5	25	0
		ー	46	E9.5	7	0
		ー	16 ^4)^	E9.5	11	0
C57BL/6	laser beam	30	ー	E9.5	10	0
		ー	35	E9.5	26	0
F1(C57BL/6x129)		18	ー	E9.5	13	0
		ー	9	E9.5	9	0
C57BL/6	microknife	31	ー	E8.5	23	0
		ー	46	E8.5	22	0
		89	ー	E9.5	65	0
		ー	88	E9.5	59	0
F1(C57BL/6x129)		47	ー	E8.5	35	0
		ー	40	E8.5	17	0
		26	ー	E9.5	2	0
		ー	28	E9.5	16	0
	Total	451	600	Total	591	0
		1051				

1) Genetic background of *cag-gfp* mice from which spleen was isolated. 2) How cell aggregates were cut into small pieces. 3) No. embryos injected with cell aggregates and transplanted into uterus of foster mothers. 4) Embryos were transplanted into foster mothers on the next day after the injection of cell aggregates.

Five hundred and sixty four embryos (210 morula and 354 blastocyst) were injected with cell aggregates cut into pieces by glass capillaries, and 294 embryos were recovered at E9.5. Ninety-two embryos (48 morula and 44 blastocyst) were injected with cell aggregates cut into pieces by laser beam, and 58 embryos were recovered at E9.5. Three hundred and ninety five embryos (193 morula and 202 blastocyst) were injected with cell aggregates cut into pieces by microknife, and 239 embryos were recovered. Seven hundred and sixty seven embryos were injected with cell aggregates derived from C57BL/6 spleen ( *B6 cag-gfp ^+/+^*), and 284 embryos with aggregates from F1 spleen between C57BL/6 and 129 ( *F1 cag-gfp ^+/-^*). Cell aggregates cut into pieces were injected into 451 morula- and 600 blastocyst-stage embryos. In total, 1,051 embryos injected with cell aggregates cut into pieces were transplanted into a foster uterus, and 591 embryos were recovered. The contribution of injected cells was judged by GFP green fluorescence in embryos (see Materials and methods). No significant contribution of the injected cells was observed in any of the 591 embryos examined. Pluripotency was not examined by injecting putative STAP cells into tetraploid embryos.

## Discussion

Two reports of unsuccessful attempts at “STAP cell” formation have been published; both judged by *oct-gfp* and pluripotent marker gene expression ( De Los Angeles *et al.*, 2015; Tang *et al.*, 2014). One of the central claims in the original reports was that the purported STAP cells had the ability to differentiate into multiple lineages, including germ cells, when placed in a normal developmental environment. However, this was not examined in these two reports. The key question is how “STAP cells” are prepared. In the original reports, the STAP cells were prepared by Haruko Obokata, while the chimera production and the establishment of two stem cells, STAP-SCs that were ES (embryonic stem)-like and FI-SCs that could differentiate into both extraembryonic and embryonic tissues, were made by Teruhiko Wakayama. Thus, Obokata is presumably the best qualified to prepare “STAP cells”. The present study focused on assessing pluripotency by chimera production using cell aggregates prepared by Obokata. Under the assay conditions reported here, we observed no evidence of pluripotency in cell aggregates she prepared herself.

I encourage readers to recognize a number of limitations in the studies, which were conducted under strict time constraints and in the face of considerable, often adversarial, media scrutiny. Unfortunately, it was not possible to receive technical advice from Teruhiko Wakayama in the chimera production reported here, and it is unclear whether or to what extent the techniques for chimera production in the present study correspond to those used in the previous studies. Previous studies also examined the pluripotency of purported STAP cells by their potency to generate teratomas in immune-deficient mice. However, more than 10 ^5 ^cells are required to form teratoma subcutaneously in the flank of an immune-deficient mouse using ES or EC (embryo carcinoma) cells, and the process takes about one month. No teratoma formation was examined in the present study, since the frequency of green fluorescent cell aggregates was low and time was limited. Teratoma formation under the kidney capsule, which also takes about two months using blastocyst embryos, was also not examined.

The more critical question is whether and to what extent the STAP cell aggregates prepared by Obokata in this trial under new experimental conditions recapitulated the STAP cell aggregates reported in the previous study. The frequency of green fluorescent cell aggregates from low pH-treated, *oct-gfp* transgenic spleen cells was 10-fold less than that in previous studies. Moreover, green fluorescence due to GFP expression cannot be distinguished from that due to autofluorescence, nor can GFP expression by reprogramming be distinguished from that due to non-specific gene expression in dying cells. The cell aggregates were not characterized *in vitro* in detail, but the following features were observed:

(1) Preliminary FACS analysis of low pH-treated, *oct-gfp* transgenic spleen cells suggested that the frequency of green fluorescent cells was very low and that the majority of surviving cells were CD45-positive after one week in culture under the conditions used in the present study. In the previous study, CD45 ^+^ cells were rare and a significant number of green fluorescent cells were observed (Figure 1c in Obokata *et al.*, 2014a).

(2) Preliminary qPCR analysis suggested that the majority of the cell aggregates generated in the present study did not express pluripotency markers, in contrast to the report of pluripotency marker expression in the previous study (Figure 2b in Obokata *et al.*, 2014a), although there were cell aggregates at a low frequency that expressed one or multiple pluripotent markers including the *oct-gfp* transgene.

(3) Preliminary immunochemical analysis suggested that most of the cell aggregates in the present study did not express pluripotency markers. In contrast to the data shown in Figure 2a of the previous study, they did not express OCT4, SSEA1, NANOG and E-CADHERIN, ( Obokata *et al.*, 2014a).

The possibility cannot be excluded that the experimental conditions used in the present study in some way differed from the previously established optimum conditions for STAP induction. It is my view that it was beyond the scope of this examination to reassign each condition; a definitive answer to the question of whether the previously used conditions for inducing the STAP phenomenon can be indeed established or not must await further study. Nevertheless, I consider it is important to report that Haruko Obokata herself failed to reproduce the reported phenomenon, in that the putative STAP cells described here were unable to contribute to any tissues in a normal developmental environment.

Another reported feature of the STAP phenomenon was that while STAP cells themselves do not proliferate, two types of stem cells could be established from them: STAP-SCs and FI-SCs. However, as Obokata had no experience with these stem cell culture, she did not undertake the establishment of the secondary stem cell types in the present study.

## Materials and methods

### Animals

C57BL/6NJcl and 129X1/SvJJmsSlc mice were purchased from CLEA Japan and Japan SLC, respectively. A transgenic mouse line harboring *gfp* under an *Oct4* promoter ( *oct-gfp*/GOF-Tg; Ohbo *et al.*, 2003) was provided by RIKEN BioResource Center (BRC) to CDB, and has been maintained in homozygous state under C57BL/6 background in CDB animal facility. A transgenic mouse line harboring *gfp* under a *CAG* promoter ( *cag-gfp* Tg; Okabe *et al.,* 1997) was provided to CDB by Masaru Okabe at Osaka University, and has been maintained in homozygous state under C57BL/6 background in CDB animal facility. Animals were housed in environmentally controlled rooms, and animal experiments were conducted under the institutional guidelines for Animal and Recombinant DNA Experiments that are consistent with ARRIVE guidelines. The experiments were approved by Institutional Animal Care and Use Committee of RIKEN Kobe Branch (Permit No., AH26-01).

### Preparation of cell aggregates

Newborn male mice of 6–8 days old were euthanized using carbon dioxide and then sterilized with 70% ethanol. Two spleens were placed in a 15 ml conical tube, minced by scissors into paste, added with 5.5 ml HBSS (GIBCO 14170), mechanically dissociated using a Pasteur pipette and strained through a cell strainer (mesh size 40 μm, FALCON 352340) into another conical tube. Five ml of Lympholyte-M (Cedarlane CL5031) was added to the bottom of the tube beneath the cell suspension, and the tube was centrifuged at 1,500 g for 20 min. The middle lymphocyte layer was transferred into another tube and centrifuged at 800 g for 10 min. The pelleted cells were suspended in 500 μl HBSS, of which 6 μl was subjected to the counting of cell number; in exchange 6 μl 200 mM ATP (SIGMA 3377) or diluted HCl (10 μl 35% HCl to 590 μl HBSS) was added to the cell suspension. The cell suspension was incubated at 37°C for 15 min in 5% CO _2_ incubator, and then centrifuged at 1,500 rpm for 15 min at room temperature. After the supernatant was removed, B27 medium (DMEM/F-12 (GIBCO 11330) supplemented with 1,000 U LIF (ESGRO 1107), 2% B-27 (GIBCO 17504) and 1 μg/ml bFGF (WAKO 060-04543) was added to the cell pellets to obtain 1×10 ^6^ cells/ml suspension; one ml of the suspension was plated in each well of a 24 well plate (FALCON 353047) and cultured at 37°C in 5% CO2 incubator for seven days to develop cell aggregates. Cell aggregates of 50–100 μm were examined for green and red fluorescence with an Olympus Fluorescent Microscope IX51 (mirror units: Olympus U-MNIBA2 to detect green fluorescence and Olympus U-MWIG2 to detect red fluorescence), and the number of candidate aggregates were counted by Haruko Obokata. Images were taken with an Olympus DP70 camera coupled with Olympus DP Controller software (version 1.2.1.108).

### Chimeric assay for pluripotency

Cell aggregates prepared by Haruko Obokata were subjected to chimera production. Cell aggregates were cut into small pieces by either glass capillary, laser beam (XY Clone: Nikko Hansen & Co., Osaka, Japan) or microsurgical knife (K-5310: FEATHER Safety Razor Co., Osaka, Japan). The pieces were injected into host embryos of either E2.5 8-cell stage or E3.5 blastocyst stage embryos of random-bred ICR (Charles River, Tokyo, Japan). Injected embryos were transplanted into the uterus of pseudopregnant females of the ICR strain. Injection of cell aggregates and transplantation of the embryos into pseudopregnant females were performed as routinely done with ES cells ( http://www2.clst.riken.jp/arg/Methods.html). Embryos were recovered at E9.5 or E8.5 and examined for the contribution of injected cells by detecting the presence of GFP-green fluorescence with LEICA fluorescence stereomicroscope M165FC (filter sets 10447407 and 10447408). E9.5 or E8.5 embryos of the *cag-gfp* transgenic line used for the preparation of cell aggregates served as positive control and wild type ICR embryos as negative control for the green fluorescence detection.

See Niwa (2016) for QPCR, immunostaining and FACS analysis.

## Data availability

The data referenced by this article are under copyright with the following copyright statement: Copyright: © 2016 Aizawa S

**Open Science Framework: Dataset: Results of an attempt to reproduce the STAP phenomenon, doi** 10.17605/OSF.IO/48f2z ( Aizawa, 2016).
