# Supplementary material for: Results of an attempt to reproduce the STAP phenomenon
Source: F1000Res. 2016 Oct 17;5:1056. Originally published 2016 Jun 1. [Version 2] doi: 10.12688/f1000research.8731.2 (PMC4995676; doi:10.12688/f1000research.8731.2)
Supplement: Peer review history

# Peer review: Referee response for version 2

**DOI:** 10.5256/f1000research.10449.r17036

**Published:** 2016-10-17

**Authors:** Austin Smith

**Affiliations:** 1 Wellcome Trust-Medical Research Council Cambridge Stem Cell Institute, and Department of Biochemistry, University of Cambridge, Cambridge, UK

I have read this submission. I believe that I have an appropriate level of expertise to confirm that it is of an acceptable scientific standard.

# Peer review: Referee response for version 1

**DOI:** 10.5256/f1000research.9395.r15460

**Published:** 2016-08-22

**Authors:** Irene de Lazaro

**Affiliations:** 1 Division of Pharmacy and Optometry, School of Health Sciences, Faculty of Biology, Medicine and Health, The University of Manchester, Manchester, UK

Two 2014 *Nature* papers authored by Obokata *et al.* described a novel protocol for the generation of pluripotent cells from somatic cells, based on their exposure to stress-triggering conditions such as mechanical disruption or acid treatment. Such process was named as stimulus-triggered acquisition of pluripotency (STAP), but the studies were soon retracted on the grounds of several confirmed experimental errors and indications of research misconduct. The present work by Dr. Aizawa describes a systematic study aiming to clarify whether STAP putative pluripotent cells – generated by Obokata under the supervision of RIKEN’s Scientific Validity Examination Team – contribute to the development of the mouse embryo and hence can be considered *bona fide* pluripotent cells.

   

 The conclusions reached in this study – that the claims for STAP cells functional pluripotency are not reproducible – are clearly supported by the data provided, since STAP cell contribution was not observed in any of the recovered embryos. The number of embryos injected with STAP cells to investigate their contribution to different tissues are satisfactorily high. In addition, the design of the study is very systematic and accounts for several potential sources of variability in the chimerism outcome (i.e. source of stress stimulus, technique utilised to cut the cell clusters before microinjection, stage of the embryos at injection and recovery time). Nevertheless, please find below a few suggestions and clarifications that in my opinion could enrich the study by Dr Aizawa.

**Experimental design:**

- In the retracted studies by Obokata *et al.*, CD45 ^+^ spleen cells were selected by FACS as the source for STAP cells generation. In the present study, sorting for CD45 was omitted, and the commercial product Lympholyte that reportedly allows specific isolation of lymphocytes was used instead. Being this an alteration of the original protocol under investigation, which may introduce differences in the nature of the starting cell population, it would be beneficial if the author could explain any reason behind this change.
- It is mentioned that the CAG-GFP transgenic mouse line utilized for chimerism experiments was different to that used by Obokata *et al.* in the previous, now retracted, studies. Was there a reason to select a different line?
- In the original Obokata *et al.* studies, clusters of STAP cells were injected into E4.5 embryos. However, in the present study the injection was performed into embryos at the E2.5 or E3.5 stage. Was this parameter changed to attempt a higher degree of chimerism? An explanation for this change in the experimental setup would also be desirable for clarity.

 

**Presentation, treatment and discussion of data:**

  

- **Frequency of cell aggregates from Oct-GFP transgenic spleen after low pH treatment (Table 1):** although it is stated in the text that “no apparent difference was found in the frequency of green fluorescence signal under either of the low-pH conditions (HCl or ATP) or genetic background of mice” this statement would be stronger if it was supported by an statistical test. Did the author conduct statistical analysis on these data?
- **Green and red fluorescence of cell aggregates:** the author seems to imply that the detected signal was a result of autofluorescence, which has been in fact pointed out by other researchers attempting to reproduce the work (Tang *et al.* 2014; De los Angeles *et al.* 2015; see last comment below). However, this is not clearly stated in the text. In addition, doubts concerning green autofluorescence could be easily vanished by using an anti-GFP antibody or by measuring the levels of GFP mRNA by qPCR or GFP protein by Western Blot. I strongly encourage the author to perform such studies if samples are still available since they would definitely clarify the issue. The inclusion in the study of cell aggregates derived from a wild-type mouse line would have also avoided this ambiguity.
- **Genetic background of mice to generate STAP cells for chimerism studies:** It is first stated in the text that the CAG-GFP mice involved in chimerism studies were bred on a C57BL/6 homozygous background. However, it is later highlighted in the text, as well as in Table 2, that both C57BL/6 abnd F1(C57BL/6x129) were included. Since those were also the backgrounds selected to maintain Oct-GFP transgenics for the cell aggregates assay, this is confusing needs clarification from the author.
- **Results included in Niwa, 2016:** the author refers several times to the results reported by Niwa (Niwa, 2016), who also investigated the reproducibility of the STAP phenomenon for the Scientific Validity Examination Team in RIKEN . It is however not clearly specified whether Niwa’s studies were conducted on the same STAP cells produced by Obokata under strict supervision. Such clarification is important, since qPCR, immunostaining and FACS data are discussed, but not shown, and the reader is directed to Niwa’s work.
- At least two other independent studies, carried out at institutions outside RIKEN, have aimed to clarify the STAP controversy (Tang *et al.* 2014, De los Angeles *et al.* 2015) and reached similar conclusions to those presented in this article. In particular, the issues on autofluorescence have been extensively scrutinized in De los Angeles *et al*. I believe that a brief discussion of the observations compiled in such studies would strengthen the present manuscript.
- **Typographical error:** “in the intestests of clarifying the scientific record” (page 2 of 8).

I have read this submission. I believe that I have an appropriate level of expertise to confirm that it is of an acceptable scientific standard.

## References

[1] TangMKLoLMShiWTYaoYLeeHSLeeKK: Transient acid treatment cannot induce neonatal somatic cells to become pluripotent stem cells. *F1000Res*.2014;3: 10.12688/f1000research.4092.1 102 10.12688/f1000research.4092.1 25075303PMC4032108

[2] De Los AngelesAFerrariFFujiwaraYMathieuRLeeSLeeSTuHRossSChouSNguyenMWuZTheunissenTPowellBImsoonthornruksaSChenJBorkentMKrupalnikVLujanEWernigMHannaJHochedlingerKPeiDJaenischRDengHOrkinSParkPDaleyG: Failure to replicate the STAP cell phenomenon. *Nature*.2015;525(7570) : 10.1038/nature15513 E6-E9 10.1038/nature15513 26399835

[3] NiwaH: Investigation of the cellular reprogramming phenomenon referred to as stimulus-triggered acquisition of pluripotency (STAP). *Sci Rep*.2016;6: 10.1038/srep28003 28003 10.1038/srep28003 27292224PMC4904271

# Response

**Published:** 2016-09-24

**Authors:** Shinichi Aizawa

**Affiliations:** RIKEN Center for Developmental Biology, Japan

Dear Dr. Irene de Lazaro,

 I thank you for your comments. The manuscript was revised incorporating your suggestions. My responses are as follows: 

  

1. There was no FACS cell sorter in the laboratory in which Obokata performed the set of supervised experiments reported here. She had previously obtained “STAP” cells using splenocytes prepared using Lympholyte-M, so we sought to determine whether she was able to repeat this in the present study. If she had succeeded, our plan was next to generate STAP cells using CD45+ cells sorted by FACS.
2. The origin of the *cag-gfp* transgenic mouse line used in the retracted *Nature* papers is unclear, and was not reported in the papers. Dr. Wakayama informed us that he generated the *cag-gfp* mouse line himself while at the University of Hawaii, but we did not make a formal investigation into this. The mouse line was no longer maintained in the animal facility of CDB and was not available to us. Alternatively, the *cag-gfp* mouse line may have been actually an *Acr/cag-gfp* mouse line (Nakanishi *et al.,* Genomics 80, 564-574 (2002)) as suggested in the report by Konno *et al* (Konno *et al.,* Nature 525,E4-5 (2015). However, we only became aware of this possibility at the time of that report, which was after the start of Obokata’s replication attempt. In any case, the *cag-gfp* mouse line reportedly used in the original STAP reports is different from the *cag-gfp *mouse line (Okabe *et al.,* 1997) we used in the present study. It is nonetheless difficult to conceive how the difference in *cag-gfp* transgene might affect the efficiency of “STAP cell” production and chimera generation.
3. In Fig. 4a of the retracted *Nature* article, the embryo being injected with “STAP” cells clearly has a zona pellucida. However, E4.5 embryos typically no longer have this structure. In the absence of zona pellucida, injection is practically impossible. We note that E0 is generally defined as 0:00 am of the day when the plug is identified, and suggest that E4.5 may be a typographic error for E3.5. Alternatively, Dr. Wakayama may have artificially delayed the development of the embryo; however, this was not reported in the retracted *Nature *paper.
4. We have now included a statistical analysis (t-test), which indicates that the efficiency of cell aggregate formation is significantly different between ATP treatment and HCl treatment in the C57BL/6 background. However, the difference is slight. We have revised the manuscript accordingly (Table 1 and page 5 in the text).
5. This study focused on the multipotency of cell aggregates generated by Obokata using a chimeric assay as this was the central feature of the reported “STAP” phenomena. Given the time constraints of this study, other data were necessarily limited, as noted in the Discussion. As it was not the focus of the present study, I cannot state definitively that the red fluorescence observed was autofluorescence, although I feel that this is highly likely. RT-PCR analysis for GFP expression showed significant expression in several aggregates, but not in others that showed green fluorescence; however, these data were preliminary at best and are not presented.
6. The effects on both cell aggregate formation and chimeric potency of the spleens’ genetic background were examined in the C57BL/6 and F1(C57BL6 x 129) background. It is well known that ES culture is strongly influenced by genetic background. Both of these backgrounds were used in the retracted *Nature *papers. I have now revised the manuscript (page 4 and page 6) to clarify this point.
7. The cell aggregates in Niwa’s report were prepared by Niwa, not by Obokata.
8. The two reports are now cited and briefly discussed (page 8–9). These works did not examine multipotency by chimeric assay, and the most important issue of the present report is that cell aggregates prepared by Obokata herself did not exhibit multipotency in chimeric assays.

 

 Best regards,

 Shin Aizawa

# Peer review: Referee response for version 1

**DOI:** 10.5256/f1000research.9395.r14102

**Published:** 2016-06-13

**Authors:** Austin Smith

**Affiliations:** 1 Wellcome Trust-Medical Research Council Cambridge Stem Cell Institute, and Department of Biochemistry, University of Cambridge, Cambridge, UK

In this paper Dr Aizawa reports the outcome of attempts to reproduce the claim that exposure to low pH can convert splenocytes into pluripotent cells, so-called STAP cells, that are capable of colonising the mouse embryo. Although the two STAP papers have now been retracted acknowledging multiple errors and misconduct, the retraction notice does not state that the results are irreproducible but only says “we are unable to say without doubt whether the STAP-SC phenomenon is real”. This study is therefore a valuable service to the community. It is unfortunate that Ms Obokata cannot be contacted. It would be desirable if she confirmed her agreement with the findings. However, I do not think there is any requirement for her to be a co-author because she carried out the work under the explicit direction and supervision of Dr Aizawa.

 The study design, results and interpretation are clearly presented. Putative STAP cell aggregates, as defined by Ms Obokata on the basis of fluorescence and/or morphology, were obtained. In a comprehensive series of micro-injections these cells were introduced into morulae or early blastocysts then transferred to recipient mice. No contribution was detected in 591 recovered embryos inspected for expression of a constitutive GFP reporter. Therefore the findings reported in the STAP papers cannot be reproduced using “STAP” cells generated by Ms Obokata in supervised conditions. This is a helpful clarification for the field.

 I have a few minor suggestions and questions that could improve the clarity of the manuscript:

- In Table 1 the heading Exp No should be changed to No. of Expts and the heading No. Cell Aggregates should be No. Fluorescent Cell Aggregates.
- It is not clear from the Table or text what proportion of aggregates showed fluorescence or whether they all did. This should be stated.
- It is stated that the source of fluorescence could not be confirmed. Were no aggregates generated from wildtype splenocytes without a reporter? How intense is the green fluorescence in aggregates compared with the Oct4-GFP level in embryos or ES cells? The text should explain that red fluorescence is autofluorescence.
- For the chimaera experiments it is stated that “cell aggregates of 50-100mM were selected by their cluster morphology by Obokata”. Can “cluster morphology” be described more precisely?
- Typographical error: “cell aggregates were one cut into small pieces”.
- Were any injected embryos examined for donor cell survival/integration prior to uterine transfer?
- Could the author comment on the limit of detection (number of cells) for chimaera contribution at the stages examined using this reporter? The method “Embryos were ….. examined for the contribution of injected cells in each organ”. I assume this was in whole mount rather than dissected organs, but this should be declared.

I have read this submission. I believe that I have an appropriate level of expertise to confirm that it is of an acceptable scientific standard.

# Response

**Published:** 2016-09-24

**Authors:** Shinichi Aizawa

**Affiliations:** RIKEN Center for Developmental Biology, Japan

Dear Dr. Austin Smith,

 I thank you for your comments. The manuscript was revised incorporating your suggestions. My responses are as follows: 

1. The headings in Table 1 have been changed as suggested.
2. All *oct-gfp* cell aggregates exhibited fluorescence to some degrees.
3. No cell aggregates were generated from wild-type splenocytes. No direct comparison was made of the intensities of green fluorescence of cell aggregates with those in *oct-gfp* embryos or ES cells. I cannot state with certainty whether the green and red fluorescence was autofluorescence. RT-PCR analysis for GFP expression showed significant expression in several aggregates, but not in others that had green fluorescence; these data were very preliminary and thus are not shown. This examination focused on the multipotency of cell aggregates generated by Obokata using a chimeric assay, since this was the central feature of the STAP phenomena. Other data were only preliminary given the time constraints under which these experiments were performed, as described in Discussion.
4. To make chimeras, cell aggregates were prepared with *cag-gfp *splenocytes, thus GFP expression or green fluorescence cannot be used as a measure for the selection of cell aggregates. For this reason, they could only be selected by cell cluster morphology. In the present study, the selection was dependent entirely on Obokata’s judgment. If she had succeeded, our plan was next to ask her to describe “cell cluster morphology” precisely.
5. Many embryos injected with *cag-gfp* cell aggregates at 8-cell stage and cultured for one day to the blastocyst stage were examined for the presence of green-fluorescent cells, and such cells were found to be present.
6. Chimeric extent was examined at E9.5 or E8.5 in whole mount. The retracted *Nature* papers show extensive colonization of the cells (Fig. 4 in the Article and Fig. 1 and Extended Data Fig. 1 in the Letter). The article reported eight chimeric embryos, showing more then 50% coat color contribution, of 48 chimeras obtained; these animals yielded “STAP”-derived offspring. This was the central finding in the now-retracted STAP reports. However, in the present study, no chimera equivalent to those in Fig. 4 in the article and Fig. 1 and Extended Data Fig. 1 in the Letter was obtained, nor were any chimeras obtained showing more than 50% contribution to coat pigmentation. Indeed, no chimera showing more than 1% contribution was obtained. I have now revised the text accordingly. We have not examined the limit of detection (minimum number of cells) with the *cag-gfp* mouse line used, since this was not the point of the present study. However, I believe it to be the case that if dozens of cells had been present together in any tissue, they would have been detectable in whole mount at E9.5 or E8.5.

Best regards,

 Shin Aizawa
